# Accuracy of Artificial Intelligence for Gatekeeping in Referrals to Specialized Care

**DOI:** 10.1001/jamanetworkopen.2025.13285

**Published:** 2025-06-03

**Authors:** Piter Oliveira Vergara, Jeronimo de Conto Oliveira, Rita Mattiello, Alfredo Montelongo, Rudi Roman, Natan Katz, Leandro Krug Wives, Dimitris Varvaki Rados

**Affiliations:** 1 Institute of Informatics, Federal University of Rio Grande do Sul, Porto Alegre, Brazil; 2 Núcleo de Telessaúde do Rio Grande do Sul (TelessaúdeRS), Federal University of Rio Grande do Sul, Porto Alegre, Brazil; 3 Post Graduate Studies Program in Epidemiology, School of Medicine, Federal University of Rio Grande do Sul (UFRGS), Porto Alegre, Brazil

## Abstract

**Importance:**

Integrating artificial intelligence (AI) technologies into gatekeeping holds significant potential, as it efficiently handles repetitive tasks and can process large amounts of information quickly.

**Objective:**

To develop and assess the accuracy of an AI model that enhances the gatekeeping process for referrals to specialized care.

**Design, Setting, and Participants:**

This diagnostic study comprised referrals from primary care to endocrinology, gastroenterology, proctology, rheumatology, and urology from a retrospective administrative database of patients in Brazil between June 2016 and April 2019. Analysis was performed between December 2022 and July 2024.

**Main Outcomes and Measures:**

The algorithm’s development and testing comprised 2 stages. Multiple AI models were initially evaluated to train and test the algorithm for categorizing referrals as authorizing or requiring additional information. Subsequently, the model’s performance was assessed against an independent set of referrals. Additionally, the current (human) evaluations of gatekeepers were evaluated against the standard. The reference standard was the consensus of 2 physicians with extensive experience. Accuracy, sensitivity, specificity, and area under the receiver operating characteristic curve (AUC-ROC) were assessed.

**Results:**

The electronic system retrieved 45 039 eligible referrals for the development stage (mean [SD] patient age, 51.9 [15.8] years; 25 458 women [56.5%]). An algorithm utilizing word embeddings and a neural network proved the most effective. In the second phase, 1750 referrals (350 for each specialty) showed a 32% authorization rate according to the reference standard. The AI model achieved an overall accuracy of 0.716 (95% IC, 0.694-0.737), with a sensitivity of 0.542 (95% CI, 0.501 to 0.582) and specificity of 0.801 (95% CI, 0.777 to 0.822). Regarding individual specialties, rheumatology exhibited the highest accuracy (0.811; 95% IC, 0.767-0.849), while proctology had the lowest (0.649; 95% IC, 0.597-0.697). The overall AUC-ROC was 0.765 (95% IC, 0.742-0.788). When compared against the consensus standard, the AI model had higher accuracy and specificity and lower sensitivity than the current approach.

**Conclusions and Relevance:**

In this diagnostic study of referral data, a novel AI model effectively distinguished between referrals that warranted immediate authorization and those that required further information with moderate accuracy; it had higher specificity and lower sensitivity than gatekeepers decisions. Implementing this AI model in the gatekeeping process should combine human judgment and AI support to optimize the referral process.

## Key Points

**Question:**

How accurate is an artificial intelligence (AI) model designed for gatekeeping referrals from primary to specialized care?

**Findings:**

In this diagnostic study of an AI model for gatekeeping that was developed, tested, and validated sequentially, the model differentiated between referrals that warranted immediate authorization and additional information with moderate accuracy and outperformed human gatekeepers by nearly 20%, primarily due to increased specificity.

**Meaning:**

These results suggest that an AI model could be integrated into the gatekeeping process for referrals from primary to specialized care, potentially improving patient triage.

## Teaser

This diagnostic study examines the accuracy of an artificial intelligence model to evaluate primary care referrals to specialty care in Brazil.

## Introduction

The global demographic shift toward an aging population, coupled with the rising burden of chronic illnesses, has led to an escalating need for specialized health care services.^1,2^ Within this context, referrals from primary care to specialists play a pivotal role, facilitating patients’ access to specialized knowledge and expertise.^1^ However, some challenges (inadequate decision-making and communication, supply-demand mismatches) can hinder the referral process.^3,4^ Prolonged waiting may lead to more advanced disease, increase health care–related anxiety, and contribute to morbidity and mortality. Addressing these challenges is crucial to optimize patient care and health care resource utilization.^3,4,5^

Managing referrals requires balancing access while controlling demand for limited resources.^3,6^ Primary care uses gatekeeping to regulate access to specialist services and manage demand. However, implementing gatekeeping measures can be complex and have potential drawbacks, including a negative impact on patient satisfaction.^7^ Nonetheless, given high demand and constraints in expanding specialist care, gatekeeping is essential, particularly in universal health care systems.

Integrating artificial intelligence (AI) technologies into clinical practice holds immense potential for transforming the health care system. These AI-driven tools can make real-time decisions that empower health care professionals and systems, especially with high data volume.^8^ They promise reduced paperwork, allowing physicians to focus on patients and clinical functions.

Despite these potential advantages, a 2020 review underscores that AI in primary care remains in early stages, with few tools currently available for implementation.^9^ Optimizing specialist referrals emerges as an area for AI usage. This study aimed to develop and evaluate an AI model to enhance the gatekeeping for primary to specialized care referrals by supporting the first evaluation of the referrals to reduce gatekeeper workload.

## Methods

### Study Design and Ethics Approval

This diagnostic study adheres to the Standards for Reporting of Diagnostic Accuracy (STARD) reporting guideline. The study utilizes retrospective data from the Rio Grande do Sul state electronic referral system. This project was approved by the institutional review boards of Hospital de Clínicas de Porto Alegre and local health authorities, with patient consent exempted because data were deidentified with minimal risk of data exposure.

### Study Context

Brazil has a universal, primary care-oriented health care system. Gatekeeping for specialized care is vital, so a 2-tiered gatekeeping structure featuring referral protocols to facilitate decision-making is used.^10^ (A translated example of a referral protocol is provided in eAppendix 1 in Supplement 1). While some protocols are objective, others are broader, considering previous treatments, symptoms, and responses, which makes direct conversion into structured referrals unfeasible.

In the initial gatekeeping stage, primary care physicians deliver frontline care to patients and, when necessary, refer to specialists. The referral process begins with physicians entering clinical information into the system, allowing second-level gatekeepers to assess compliance with protocol criteria.

The second gatekeeping instance encompasses regulation centers staffed by administrative personnel and physicians (gatekeepers).^11^ Gatekeepers assess referral information based on protocols and request additional details when needed. The electronic system allows for several rounds of discussion until final decision; for this study, only initial data from the referral were used. Gatekeepers ensure equitable resource allocation and may reduce waiting times, while their feedback provides continuous education for primary physicians.^12,13^ Despite these advantages, improper use of the additional decision step may delay care, create inequities, and miss educational opportunities.

This study included gatekeeping system data of Rio Grande do Sul State, where TelessaúdeRS provides remote consultations, connecting primary care providers and telehealth services.^13^ This initiative improves primary care efficacy.

### Participants and Data Extraction

This study used 2 independent datasets. The first consisted of referrals from selected specialties (endocrinology, gastroenterology, proctology, rheumatology, and urology) between June 2016 and April 2019. These referrals were performed by upstate primary care clinics to specialized care in the capital city (Porto Alegre). The selection of specialties was based on several factors, including long waiting times, established referral protocols for many years, supply and demand imbalance, and their representation within clinical and surgical domains. This first dataset was used for algorithm development. Referrals were excluded if they lacked a gatekeeper’s decision, involved patients under age 18 years, or were assessed without protocols (ie, untrained gatekeepers). In the first database, the referrals were randomly divided into 2 groups, maintaining the proportion of specialties and authorization: 80% comprised the training data, and 20% formed the testing data.

A second random sample of referrals was extracted from the electronic system. These referrals were performed by primary care clinics in Porto Alegre between January and December 2019 and are handled by a different regulation center. Despite being a different center with a separate group of trained gatekeepers, the same state-approved protocols apply. This additional dataset served as an independent validation to assess the accuracy of the AI model against a reference standard. Primary physicians performed the referral by entering clinical data (and expectations) in a free-text field, which was extracted from the electronic system. Demographic variables (sex and age) were used to characterize the sample, but were not utilized in the model.

### Study Procedures

As detailed in later sections, the AI model’s development and testing occurred in 2 stages. The first focused on developing candidate algorithms for referral classification using the first dataset. After selecting the best-performing algorithm, the second phase validated it against a reference standard with an independent sample from the second dataset.

### Development and Initial Testing of the AI Model

The goal was to select the most accurate model while ensuring simplicity and low computational demand, as it would run on a public data enterprise with limited capacity. Thus, we designed the model with algorithms and hyperparameters balancing complexity and accuracy. The algorithm’s development involved text preprocessing of the clinical text field. We tested prediction with decision trees, support vector machines, and neural networks combined with different techniques for feature selection, considering their impact on algorithm’s results. Also, in the neural network, number, type and order of layers, number of neurons, and other hyperparameters were manipulated. No a priori features (ie, words) were selected. These algorithms were trained to categorize referrals as *authorizing* or *requesting additional information* in the training data (80% of the first dataset), using the decision of the regulation center as the reference. The AI model development and selection is detailed in eAppendix 2 and eFigure 1 in Supplement 1. Each model was then evaluated in the testing data (20% of the first dataset) by calculating the area under the receiver operating characteristic curve (AUC-ROC). We used Shapley Additive Explanations (SHAP) tool to explore explainability of the algorithm (eAppendix 2 in Supplement 1).

### Reference Standard and Validation in the Independent Dataset

For the last stage, we created a reference standard as an external evaluation of the second dataset (referrals from Porto Alegre primary care). The AI model selected in the previous phase was compared against the reference standard, allowing for an assessment of both AI and gatekeeper (human) accuracy. This approach enabled a direct comparison of both methods against a single comparator, providing insights into their performance and integration potential.

The referrals from Porto Alegre were evaluated by the referral center physicians. Additionally, 2 physicians (J.C.O. and D.V.R.) independently and masked evaluated each referral from this sample. Both are certified internal medicine physicians with 5 years of regulatory experience, including remote consultations for primary care physicians and protocol development. This evaluation adhered to the regulation process protocols. In cases of discordance, discussion and consensus were employed to arrive at a final decision; this final agreement was the reference standard for the study. Concordance was assessed and Cohen κ was calculated.

### Sample Size

All referrals fulfilling the inclusion criteria were included for the first dataset. For the second dataset we calculated a sample size of 313 referrals considering that approximately 35% of the referrals are authorized, with a sensitivity and specificity of 0.8 and a 0.15 margin using the Wald method. As such, we randomly selected 350 referrals for each specialty.

### Statistical Analysis

The primary analysis was the AI model’s diagnostic performance (accuracy, sensitivity, and specificity, as well as AUC) against the reference standard. Overall accuracy expresses correct classifications (both true positives and true negatives) proportion and was the preferred summary effect for reporting the results. Current decisions from the regulation center were also compared against the consensus. Finally, the diagnostic performance of the current method and the AI model were compared. When 2 methods’ accuracies are subtracted, it represents the percentage of individuals correctly (positive values) or incorrectly (negative values) reclassified in a single metric, which is also known as the absolute net reclassification index.

The ROC and precision-recall curves and AUC analysis were possible only for the AI model because its response is probabilistic. For every AI prediction, the response is a probability from 0 to 1; such information is unavailable for human decisions (which is only categorical). We set the threshold to include in the class (ie, authorizing) to 50% and explored the performance at different thresholds (60%, 70%, and 80%).

Diagnostic metrics of different methods were compared and a 2-sided *P* < .05 was considered statistically significant. Data are presented as mean results with 95% CIs. Analyses were conducted with all referrals aggregated and for each specialty individually and were performed with the Scikit-learn and SHAP python packages and MedCalc.^14,15,16^

## Results

**Figure 1.**
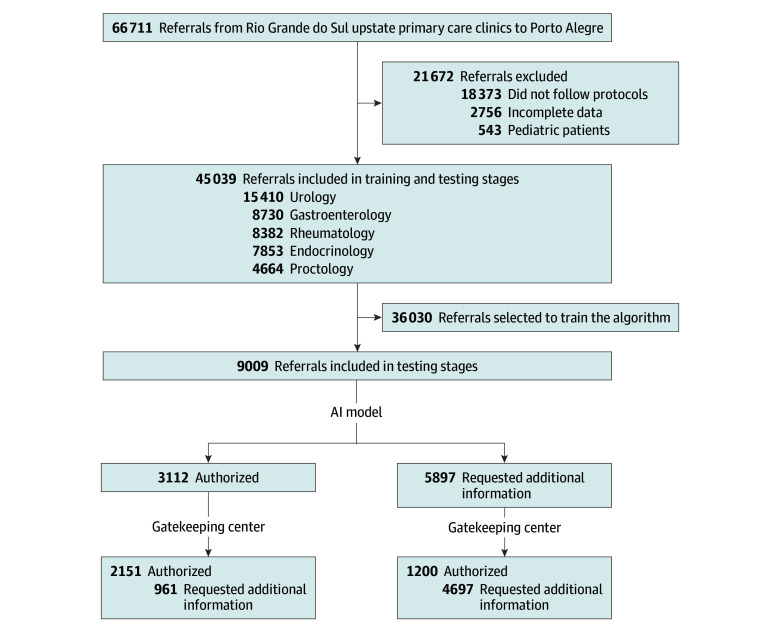
Study Flow Diagrams for the First Dataset (Training and Testing)

Initially, 66 711 referrals were extracted from the electronic system. Of those, 18 373 did not adhere to the protocols (from 20 different untrained gatekeepers), 2756 lacked initial decision, and 543 referrals were related to pediatric patients (Figure 1). A total of 45 039 cases (from 61 gatekeepers) were included in the first dataset for training (80%) and testing (20%). The mean (SD) patient age was 51.9 (15.8) years, and 25 458 (56.5%) were female. Overall, 16 757 referrals (37%) were authorized, and the remainder required additional information in the first evaluation.

**Figure 2.**
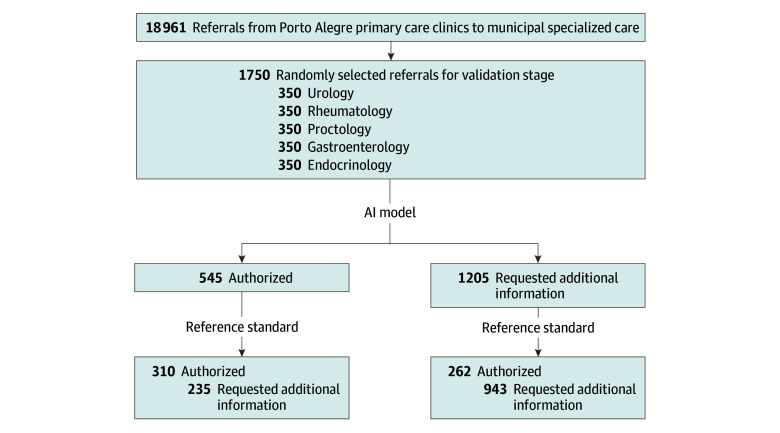
Study Flow Diagrams for the Second Dataset (Independent Validation)

For the validation dataset, 18 961 referrals were extracted and 1750 were randomly selected (350 per specialty) (Figure 2). Mean (SD) patient age was 58.6 (16.6) years; 1062 (60.7%) were female. In this final sample, 572 referrals (32.7%) of the referrals were authorized. In the standard reference evaluation, concordance between reviewers was high (79.8%), with moderate agreement (Cohen κ = 0.54).

The initial models, based on support vector machines, achieved sensitivity and specificity between 0.6 and 0.8 in the testing set. Given this performance, we opted for testing neural networks. These last models consistently achieved AUC-ROC greater than 0.75, with the best reaching 0.83 (eAppendix 2 and eFigure 1 in Supplement 1).

**Figure 3.**
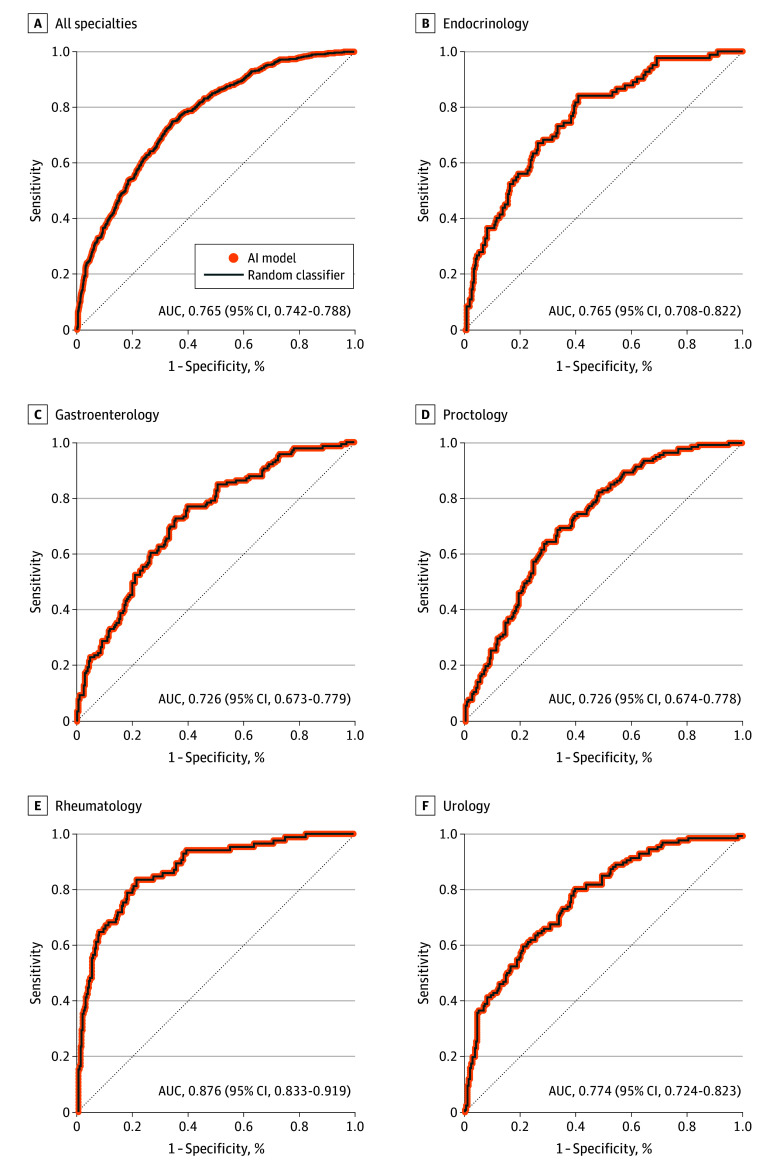
ROC Curves Comparing the AI Model With the Reference Standard, Overall and by Specialty

The AI model exhibited an overall accuracy of 0.716 (95% CI, 0.694-0.737), sensitivity of 0.542 (95% CI, 0.501-0.582), and specificity of 0.801 (95% CI, 0.777-0.822) (Table 1). Regarding individual specialties, the specificity was consistently higher than sensitivity. Specificity ranged from 0.760 (95% CI, 0.700-0.811) for urology to 0.842 (95% CI, 0.793-0.881) for rheumatology, and sensitivity ranged from 0.369 (95% CI, 0.293-0.451) for proctology to 0.718 (95% CI, 0.615-0.803) for rheumatology. The AUC-ROC for all specialties was 0.765 (95% CI, 0.742-0.788) (Figure 3). Gastroenterology and proctology showcased the lowest AUC-ROC, and rheumatology demonstrated the highest (Figure 3; eTable 1 in Supplement 1). The precision-recall curve showed that the AI model’s precision in identifying correct referrals (positive predictive value) trades off with sensitivity (recall); so increasing sensitivity would significantly raise false positives (eFigure 2 in Supplement 1).

**Table 1.** Diagnostic Performance of AI Model and Current Regulation Methods Compared With the Reference Standard

Specialty	Mean (95% CI)			*P* value
	AI model	Current method	Difference	
**Specificity**				
All specialties	0.801 (0.777 to 0.822)	0.340 (0.313 to 0.367)	0.461 (0.425 to 0.495)	<.001
Endocrinology	0.780 (0.727 to 0.826)	0.399 (0.342 to 0.459)	0.381 (0.301 to 0.454)	<.001
Gastroenterology	0.782 (0.722 to 0.833)	0.592 (0.525 to 0.657)	0.190 (0.102 to 0.274)	<.001
Proctology	0.837 (0.782 to 0.882)	0.225 (0.173 to 0.286)	0.612 (0.529 to 0.680)	<.001
Rheumatology	0.842 (0.793 to 0.881)	0.162 (0.122 to 0.211)	0.680 (0.610 to 0.736)	<.001
Urology	0.760 (0.700 to 0.811)	0.347 (0.287 to 0.411)	0.413 (0.325 to 0.491)	<.001
**Sensitivity**				
All specialties	0.542 (0.501 to 0.582)	0.897 (0.869 to 0.919)	−0.355 (−0.401 to −0.306)	<.001
Endocrinology	0.561 (0.453 to 0.663)	0.890 (0.806 to 0.942)	−0.329 (−0.448 to −0.195)	<.001
Gastroenterology	0.525 (0.443 to 0.606)	0.835 (0.765 to 0.888)	−0.310 (−0.408 to −0.202)	<.001
Proctology	0.369 (0.293 to 0.451)	0.894 (0.833 to 0.935)	−0.525 (−0.610 to −0.422)	<.001
Rheumatology	0.718 (0.615 to 0.803)	0.941 (0.871 to 0.976)	−0.223 (−0.332 to −0.112)	<.001
Urology	0.624 (0.537 to 0.704)	0.944 (0.890 to 0.974)	−0.320 (−0.412 to −0.223)	<.001
**Accuracy**				
All specialties	0.716 (0.694 to 0.737)	0.522 (0.498 to 0.545)	0.194 (0.162 to 0.225)	<.001
Endocrinology	0.729 (0.680 to 0.773)	0.514 (0.462 to 0.566)	0.214 (0.144 to 0.283)	<.001
Gastroenterology	0.680 (0.630 to 0.727)	0.689 (0.638 to 0.735)	−0.009 (−0.075 to 0.060)	.80
Proctology	0.649 (0.597 to 0.697)	0.494 (0.442 to 0.546)	0.155 (0.082 to 0.226)	<.001
Rheumatology	0.811 (0.767 to 0.849)	0.351 (0.303 to 0.403)	0.460 (0.392 to 0.521)	<.001
Urology	0.711 (0.662 to 0.757)	0.560 (0.508 to 0.611)	0.151 (0.080 to 0.220)	<.001

Abbreviation: AI, artificial intelligence.

The comparison of the AI model and the current gatekeeping shows an absolute accuracy increase of 0.194 (95% CI, 0.162-0.225; *P* < .001) (Table 1). This means a correct reclassification of 19 out of 100 persons evaluated with AI (eTable 2 in Supplement 1). The accuracy gains varied, ranging from 0.460 in rheumatology (95% CI, 0.392 to 0.521; *P* < .001) to no difference in gastroenterology (−0.009; 95% CI, −0.075 to 0.060; *P* = .80) (Table 1).

The AI model’s positive and negative predictive values were 0.568 (95% IC, 0.526-0.609) and 0.782 (95% IC, 0.758-0.805), respectively, compared with 0.397 (95% IC, 0.370-0.424) and 0.871 (95% IC, 0.837-0.899) for the current method. This shows a more balanced predictive capacity for the algorithm (eTable 3 in Supplement 1). The absolute results are detailed in eTable 4 in Supplement 1.

By adjusting the response threshold, we explored the AI model’s performance across different scenarios. Increasing the threshold improved specificity while reducing sensitivity, with no significant impact on accuracy (Table 2). The SHAP analysis indicated that clinical terms were the main determinants of the algorithm (eFigure 3 and 4 in Supplement 1).

**Table 2.** Diagnostic Performance of AI Model by Different Decision Triggers Compared With the Reference Standard

Specialty	Decision trigger threshold		
	60%	70%	80%
**Specificity, mean (95% CI)**			
All specialties	0.868 (0.848-0.886)	0.928 (0.912-0.942)	0.971 (0.961-0.980)
Endocrinology	0.854 (0.807-0.892)	0.917 (0.879-0.945)	0.955 (0.923-0.974)
Gastroenterology	0.848 (0.794-0.890)	0.938 (0.898-0.964)	0.971 (0.939-0.987)
Proctology	0.909 (0.862-0.941)	0.961 (0.926-0.981)	0.995 (0.974-0.999)
Rheumatology	0.909 (0.869-0.938)	0.947 (0.913-0.968)	0.984 (0.962-0.994)
Urology	0.817 (0.762-0.863)	0.880 (0.831-0.916)	0.955 (0.920-0.976)
**Sensitivity, mean (95% CI)**			
All specialties	0.419 (0.379-0.460)	0.318 (0.281-0.357)	0.194 (0.163-0.228)
Endocrinology	0.451 (0.347-0.555)	0.365 (0.269-0.473)	0.268 (0.183-0.372)
Gastroenterology	0.352 (0.277-0.434)	0.230 (0.167-0.306)	0.143 (0.094-0.211)
Proctology	0.234 (0.171-0.309)	0.120 (0.075-0.183)	0.070 (0.038-0.124)
Rheumatology	0.647 (0.541-0.740)	0.552 (0.447-0.654)	0.294 (0.207-0.397)
Urology	0.528 (0.441-0.613)	0.448 (0.363-0.535)	0.272 (0.201-0.355)
**Accuracy, mean (95% CI)**			
All specialties	0.721 (0.700-0.742)	0.729 (0.707-0.749)	0.717 (0.696-0.738)
Endocrinology	0.760 (0.712-0.801)	0.788 (0.743-0.828)	0.794 (0.749-0.833)
Gastroenterology	0.651 (0.600-0.699)	0.657 (0.606-0.705)	0.642 (0.591-0.691)
Proctology	0.637 (0.585-0.685)	0.622 (0.571-0.672)	0.622 (0.571-0.672)
Rheumatology	0.845 (0.804-0.880)	0.851 (0.810-0.885)	0.817 (0.773-0.854)
Urology	0.714 (0.665-0.759)	0.725 (0.676-0.769)	0.711 (0.662-0.756)

Abbreviation: AI, artificial intelligence.

## Discussion

The AI model showed a moderate performance in distinguishing referrals needing authorization or additional information. It slightly outperformed gatekeepers, mainly by a lower false positive rate. Human evaluation identified unnecessary referrals more precisely (lower false negative rate). The model’s moderate performance was due to limited sensitivity.

Its reasonable false positive rate allows the algorithm to be used as a first-line evaluator for the referral. This configuration could reduce gatekeeper workload by prescreening referrals, with regulation centers reviewing only triaged cases. Of 72 000 monthly referrals in Rio Grande do Sul, most are reviewed by gatekeepers. The algorithm could immediately authorize 22 000, streamlining specialist consultations and reducing workload. This would lead to an elevated number of inappropriate approvals; however, this proportion would be lower than the current method (higher positive predictive value). Implementation could balance supply and demand by reducing incorrect authorizations and potentially lowering costs. Moreover, it is a conservative strategy, as errors would lead to unnecessary specialist consultations rather than delaying or limiting necessary care—a key concern in primary care gatekeeping systems.^17^

The algorithm produces more false negatives, but in this context, a negative decision leads to further information exchange rather than missing a diagnosis. This would require referring physicians to perform more reevaluations; however, in 4 out of 5 cases, the request would be justified. Expected benefits include reduced gatekeeper workload, fewer incorrect authorizations and unnecessary in-person consultations. However, this requires more information requests from primary care physicians. For patients, it may lead to shorter wait times and more reevaluations in primary care.

Adjusting the decision threshold is crucial. Raising it reduces false positives but requires balance. In severe diseases, more false positives may be justified to prevent delays. For resource-limited settings, higher thresholds may reduce unnecessary approvals but increase gatekeeper and primary care workload.

The patients whose referrals were directed to human review could be assessed for eligibility for an alternative to face-to-face care, such as primary care settings with remote support (provider-to-provider consultation).^13^ This approach optimizes resource allocation and improves health care efficiency, reflecting the growing interest in AI for primary care. Most studies of AI for primary care focus on diagnosing and classifying diseases.^18^ Only 3 studies^19,20,21^ focus on the health care system, which reinforces the innovative use of AI presented in this study.

Despite this optimistic scenario, while implementing AI tools in health care, administrators must remember that “an inadequately prepared physician may make mistakes one patient at a time, a faulty algorithm could affect larger numbers of patients.”^22^ For example, racial bias is a known risk in large administrative datasets.^23^

To our knowledge, only 1 prior study evaluated AI for primary care gatekeeping, focusing on otorhinolaryngology with supervised methods, achieving 0.538 accuracy on the same dataset.^24^ In comparison, our model achieved higher accuracy across all specialties, using a larger database, testing unsupervised methods, and validating against a standardized reference.

There are various unanswered topics. Continuously updating and recalibrating the model with real-time data are essential for maintaining performance. This study suggests that AI can support gatekeeping and serve as a foundation for broader use, but its application in different health care systems, locals, or specialties requires validation of this result with curated data and ongoing monitoring. Another unexplored aspect is the model’s varying performance across specialties. For example, its better performance in rheumatology may stem from the distinct clinical features and diagnostic tests of severe autoimmune diseases, which differentiate them from benign cases.

The above listed impacts on the health care system remains speculative. A prospective, ideally randomized, study is needed to assess integration with gatekeepers, safety and effectiveness of the algorithm. To address this, recently we have partnered with the local health authority and are designing a prospective trial.

### Limitations

Our study has several limitations. The ground truths for this study were derived from medical records, which may introduce inaccuracies due to errors, inconsistencies, or missing information. Such limitations could bias model predictions. SHAP analysis showed that the most relevant features were disease-related, with no single term dominating predictions, suggesting good model explainability based on clinical data. While this indicates a low risk of bias, the input data—provided by physicians—may still carry undetected (implicit) bias.

The concordance approach we choose has both strengths and limitations. It provided a curated reference, including records of complex cases, for comparing AI and the current method, highlighting their advantages and limitations. However, despite 80% concordance, the κ index was moderate, and other factors like costs and supply-demand balance were not considered.

The study utilized data from a single-state health care system and limited specialties. An external validation with an independent dataset would enhance external validity of results. Lack of detailed clinical and sociodemographic data also limits the understanding of applicability. The choice of the threshold for approval is debatable, once gatekeepers’ thinking process is unknown. We chose 50% to reflect the binary nature of decisions, but this limit is arbitrary. To address this and inform decisions, we explored other thresholds.

The exclusion of a significant sample portion can be a source of bias. Including only trained gatekeepers minimized standardization issues. However, we cannot determine whether excluding specific gatekeepers affected the findings. Nevertheless, the large number of professionals involved suggests that individual decision patterns had minimal impact.

AI is rapidly evolving, with new algorithms emerging constantly. While we tested various approaches for classifying referrals, models like transformers, reinforcement learning, and fine-tuned deep learning were not included. Maintaining low computational demand is key, but future research should explore more advanced tools for gatekeeping.

## Conclusions

In this diagnostic study of referral data, the AI model performance was moderate, with clear gains in specificity compared with the current approach when both were compared with a consensus reference standard. As referral authorization prevalence with initial data is approximately 30%, implementation of this model should focus on its positive predictive value. Threshold adjustments may improve correct authorizations. This strategy can reduce workload, allowing health care professionals to concentrate on referrals that require clinical review—an area where our study shows that gatekeepers outperformed the AI model.

## Notes

Supplement 1. **eAppendix 1.** An Example of a Referral Protocol–Hyperprolactinemia **eAppendix 2.** Model Development Description **eFigure 1.** Schematic Presentation of Model Development **eFigure 2.** Precision-Recall Curves for the AI Model Compared to the Reference Standard Overall and for Each Specialty **eFigure 3.** Top 10 Features (Terms) With the Highest Predictive Weight SHAP Analysis for the Final AI Model, by Specialty **eFigure 4.** Examples of Referrals with the Highest and Lowest Probability of Approval in the Dataset **eTable 1.** Area Under the Curve for the Artificial Intelligence Model in General and According to Specialties **eTable 2.** Net Reclassification Index (NRI) Between AI Model and Current Regulation Methods **eTable 3.** Positive and Negative Predictive Values of AI Model and Current Regulation Methods **eTable 4.** Absolute Results of the Artificial Intelligence Model and Current Regulation Methods

Supplement 2.
Data Sharing Statement
