# Supplementary material for: Accuracy of Artificial Intelligence for Gatekeeping in Referrals to Specialized Care
Source: JAMA Netw Open. 2025 Jun 3;8(6):e2513285. doi: 10.1001/jamanetworkopen.2025.13285 (PMC12134955; doi:10.1001/jamanetworkopen.2025.13285)
Supplement: Supplement 1. — eAppendix 1. An Example of a Referral Protocol–Hyperprolactinemia eAppendix 2. Model Development Description eFigure 1. Schematic Presentation of Model Development eFigure 2. Precision-Recall Curves for the AI Model Compared to the Reference Standard Overall and for Each Specialty eFigure 3. Top 10 Features (Terms) With the Highest Predictive Weight SHAP Analysis for the Final AI Model, by Specialty eFigure 4. Examples of Referrals with the Highest and Lowest Probability of Approval in the Dataset eTable 1. Area Under the Curve for the Artificial Intelligence Model in General and According to Specialties eTable 2. Net Reclassification Index (NRI) Between AI Model and Current Regulation Methods eTable 3. Positive and Negative Predictive Values of AI Model and Current Regulation Methods eTable 4. Absolute Results of the Artificial Intelligence Model and Current Regulation Methods [file jamanetwopen-e2513285-s001.pdf]

Supplemental Online Content

Vergara PO, de Conto Oliveira J, Mattiello R, et al. Accuracy of artificial intelligence for gatekeeping in referrals to specialized care. *JAMA Netw Open.* 2025;8(6):e2513285. doi:10.1001/jamanetworkopen.2025.13285

eAppendix 1. An Example of a Referral Protocol–Hyperprolactinemia

eAppendix 2. Model Development Description

eFigure 1. Schematic Presentation of Model Development

eFigure 2. Precision-Recall Curves for the AI Model Compared to the Reference Standard Overall and for Each Specialty

eFigure 3. Top 10 Features (Terms) With the Highest Predictive Weight SHAP Analysis for the Final AI Model, by Specialty

eFigure 4. Examples of Referrals with the Highest and Lowest Probability of Approval in the Dataset

eTable 1. Area Under the Curve for the Artificial Intelligence Model in General and According to Specialties

eTable 2. Net Reclassification Index (NRI) Between AI Model and Current Regulation Methods

eTable 3. Positive and Negative Predictive Values of AI Model and Current Regulation Methods

eTable 4. Absolute Results of the Artificial Intelligence Model and Current Regulation Methods

This supplemental material has been provided by the authors to give readers additional information about their work.

© 2025 Vergara PO et al. *JAMA Network Open.*

eAppendix 1. An example of a referral protocol - hyperprolactinemia.

Clinical Conditions Requiring Urgent/Emergency Referral:

- Patients with hyperprolactinemia and suspected acute optic chiasm involvement (visual impairment).

Clinical Conditions Requiring Referral to Endocrinology:

- Patients with hyperprolactinemia after exclusion of secondary causes in primary care (*see below - supporting information*).
- Patients with persistent hyperprolactinemia after discontinuation of interfering medication (*see below - supporting information*).

Minimum Required Information for Referral:

- Signs and symptoms (especially galactorrhea, menstrual cycle irregularities, and sexual dysfunction).
- Prolactin test result with date.
- Results of the investigation for secondary causes of hyperprolactinemia (*see below - supporting information*).
- Imaging report attached (preferably a contrast-enhanced MRI of the sella turcica) or a full description of the results with date (if available).
- Teleconsultation reference number, if the case was discussed with TelessaúdeRS.

© 2025 Vergara PO et al. *JAMA Network Open*.

Supporting information - initial evaluation of hyperprolactinemia in primary care.

Normal Values and Causes of Hyperprolactinemia

Normal values are considered up to 20 ng/mL in men and 30 ng/mL in non-pregnant women. Levels below 25 ng/mL typically rule out hyperprolactinemia. The stress from venipuncture can cause slight increases in prolactin levels (generally below 40 ng/mL). Therefore, if an initial prolactin level is mildly elevated, the test should be repeated. Despite the extensive list of hyperprolactinemia causes, values between 200 and 250 ng/mL are highly suggestive of a prolactin-producing pituitary adenoma (prolactinoma).

Causes of hyperprolactinemia

Group	Description
Physiological Causes	Pregnancy and breastfeeding (main causes), physical exercise, stress, intercourse, breast manipulation, and sleep.
Pharmacological Causes	The most common non-physiological cause of elevated prolactin. Associated medications include: antidepressants (clomipramine, amitriptyline, citalopram, fluvoxamine, paroxetine), antipsychotics (chlorpromazine, levomepromazine, haloperidol, risperidone, quetiapine, olanzapine, sulpiride), antihypertensives (methyldopa, verapamil), estrogens, gastrointestinal agents (domperidone, metoclopramide, cimetidine, ranitidine), opioids and cocaine, and protease inhibitors. Most drugs cause modest increases in prolactin (25–100 ng/mL), except metoclopramide and risperidone, which can lead to levels above 200 ng/mL.
Pathological Causes	Pituitary tumors that produce prolactin (most common), infiltrative hypothalamic-pituitary disorders, systemic diseases (primary hypothyroidism, primary adrenal insufficiency, polycystic ovary syndrome, cirrhosis, renal failure, systemic lupus erythematosus, anorexia nervosa, seizures).
Idiopathic	A diagnosis reserved for patients without an obvious cause of hyperprolactinemia. In most cases, this is likely due to very small microadenomas that are not visible on MRI.

Approach to the patient

- Suspected medication-induced cause: Discontinue or adjust the medication for one week (if possible) and repeat the prolactin test. If levels normalize, investigation can be stopped, confirming a drug-induced cause.
- If a drug-related cause is ruled out, request the following tests:
  - Pregnancy test (for all women of reproductive age).
  - TSH.
  - Renal function.
  - Liver transaminases.
- Asymptomatic patients with hyperprolactinemia: Request macroprolactin testing. Macroprolactin is a complex of prolactin molecules aggregated with immunoglobulin G, with high molecular weight but low biological activity. Although total prolactin levels may appear elevated in blood tests, macroprolactinemia typically has minimal clinical impact and does not require specific management.
- Suspected pituitary adenoma (if previous investigations are negative): If available, perform a contrast-enhanced MRI of the sella turcica.

© 2025 Vergara PO et al. *JAMA Network Open*.

eAppendix 2. Model development description

The model was developed by the researchers with informatics training. The initial premise was to select the most accurate model while maintaining simplicity and low computational demand. This rationale comes from the fact that, if implemented, this AI model will be hosted by the public processing data enterprise with limited processing capacity, as it provides computing support for a large number of public services from *Porto Alegre* and *Rio Grande do Sul*. As such, overall, we opted for simplified algorithms.

Therefore, the initial versions of the algorithm were based on classic predictors, such as support vector machines and decision trees. After that, using the best results in terms of ROC-AUC obtained with these classical machine learning algorithms as a baseline value, we explored a set of different neural network versions (deep learning). In this process (schematically presented in Supplementary Figure 1), we tested combinations of different tools for each step. Each combination was trained and tested in the first dataset (as presented in the main text). A difference in AUC of 0.1 was considered relevant in the comparison between models; we opted to perform no formal statistical testing to compare them.

In the first moments of the testing, we started with “classic” algorithms, such as support vector machine, random tree, and random forest (eFigure 1A). In this approach, we used several preprocessing techniques (such as lemmatization, removal of numbers, stopwords, and diacritics, and stemming) in different combinations. We also tested vectorization with frequency-inverse document frequency (TF-iDF) and word count, and representation with bag of N-grams and bag of words. Resampling, feature selection, and feature transformation were also modulated.

For this initial AI model, it achieved an AUC of 0.81, and the final architecture was:

- preprocessing the input texts with lowercase conversion, removal of diacritics and stopwords, and stemming;
- vectorizing the preprocessed texts with the TF-iDF technique;
- oversampling the resulting vectors (with KMeans SMOTE algorithm) to address the imbalance of classes;
- using a chi-squared test, choosing the best 2.000 features of those resampled;
- inputting these selected features into a Support Vector Machine predictor.

Later, we tested whether a neural network model could provide better results. In the neural networks, we always included a word embedding layer, aiming to take advantage of the context and semantics of the words in the texts. Besides testing an existing word embedding (which we also tried to expand with the corpus of this project), we worked to produce a custom one using our corpus. To do this, we tested preprocessing techniques similar to those previously applied and used the resulting texts as input to generate different word embedding vectors. We tested combinations of SkipGram and continuous bag of words (CBOW) along with FastText and Word2Vec algorithms, using different size windows and vector sizes. Each embedding was evaluated by the ROC-AUC obtained using it as the first layer in a fixed configuration neural network. At the end of these tests, we selected an embedding that uses the FastText algorithm in SkipGram mode, with a 5 token window and 300 dimensions.

Regarding network types, we explored Long Short Term Memory (LSTM) and Gateway Reccurent Units (GRU). For their hyperparameters, we tried different batch sizes (32, 64, 128, and 256), optimizers (AdaDelta, Adam, RMSProp), and dropouts (from 0.1889 up to 0.5). Also, different topologies were explored, including 4 and 8 layers and different layer types and orders. In line with our objective, whenever 2 architectures had similar performance, we chose the one with the simplest architecture.

At the end of this process, the final neural network model achieved an AUC of 0.83 in the testing dataset. It was constructed as follows:

- a preprocessing step prepares the text, converting it to lowercase, removing diacritics, numbers, special characters, stopwords, and applying stemming;
- the preprocessed text is then input into the neural network, which has the custom word embedding as first layer;
- the resulting vector goes through the next layer, which is a GRU (of 32 neurons with a dropout of 0.2333);

© 2025 Vergara PO et al. *JAMA Network Open*.

- finally, a sigmoid function produces the estimate on the output layer.

Despite the performance similarities (0.81 vs. 0.83), we opted for the neural network, as it used more recent techniques, did not lose performance in the holdout testing (which was the case for the SVM classifier), and had marginally better prediction accuracy without excessive processing demand. We expected these characteristics to be relevant for the final phase of the study. However, similar results are achievable with other model architectures.

At last, we conducted a SHapley Additive exPlanations (SHAP) analysis to assess which features (i.e., groups of terms) contributed most to the model's predictions. SHAP, based on game theory, quantifies how much each feature influences a given prediction compared to the model's average output. In this context, SHAP values help explain individual classification decisions and identify potential sources of bias. A positive SHAP value indicates that a term contributed to referral authorization, while a negative value suggests association with a request for additional information. These values are calculated for each referral and can be aggregated to assess global model behavior.

eFigure 3 presents the most influential terms by specialty (translated from Portuguese), while eFigure 4 shows examples of referrals with the highest and lowest predicted probabilities of approval, illustrating how the model weighs specific features in individual cases.

© 2025 Vergara PO et al. *JAMA Network Open*.

eFigure 1. Schematic presentation of model development. Within each procedure, the tool presented in bold was selected for the final AI model, and the non-bold alternatives were tested and compared relative to the chosen tool. (A) represent the process for "classical" machine learning techniques; (B) represent the process for neural networks.

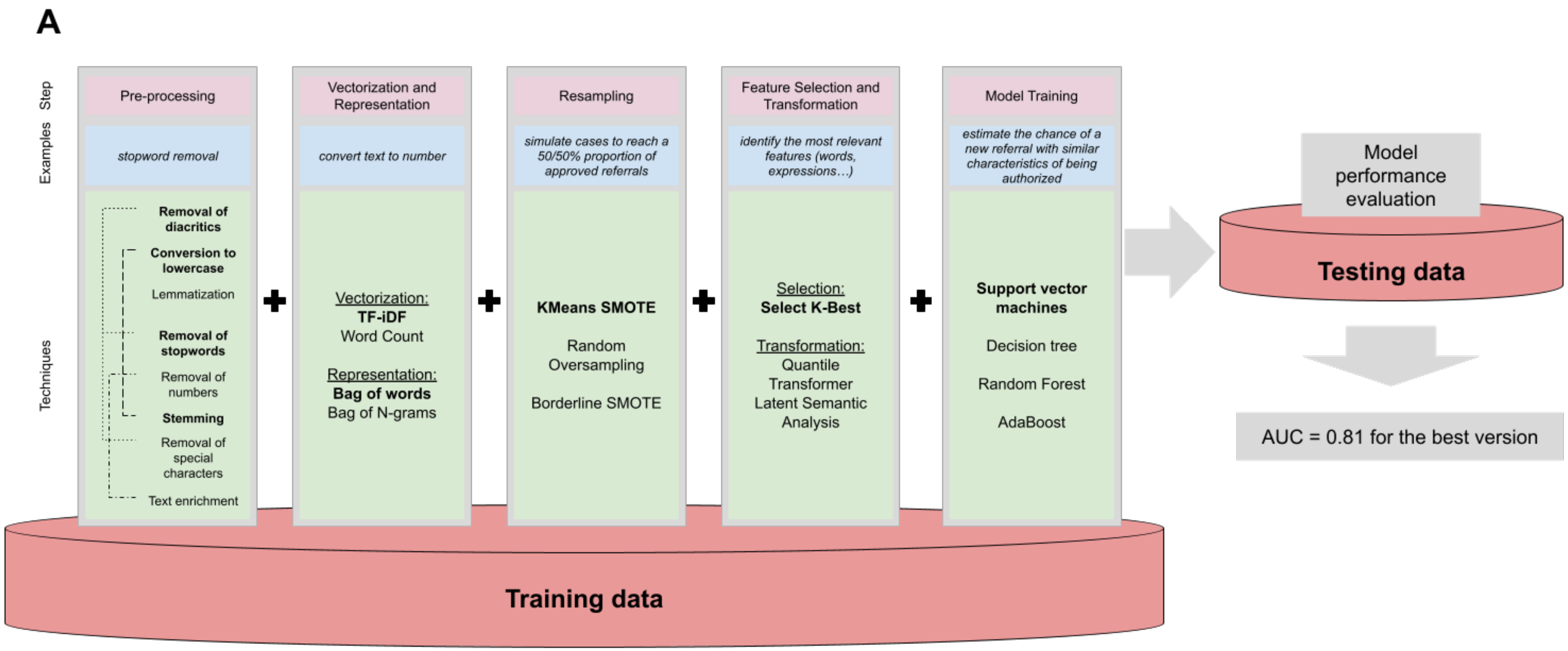

© 2025 Vergara PO et al. *JAMA Network Open*.

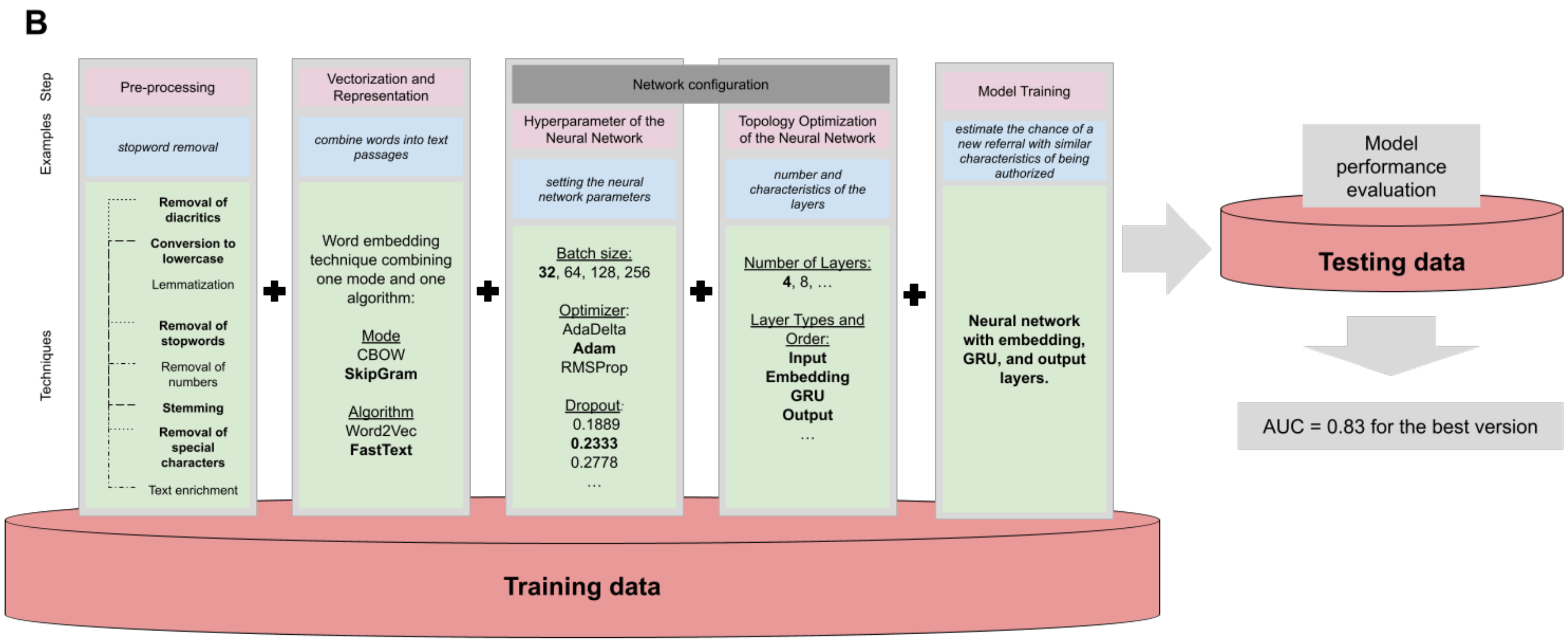

© 2025 Vergara PO et al. *JAMA Network Open*.

eFigure 2. Precision-recall curves for the AI model compared to the reference standard overall and for each specialty.

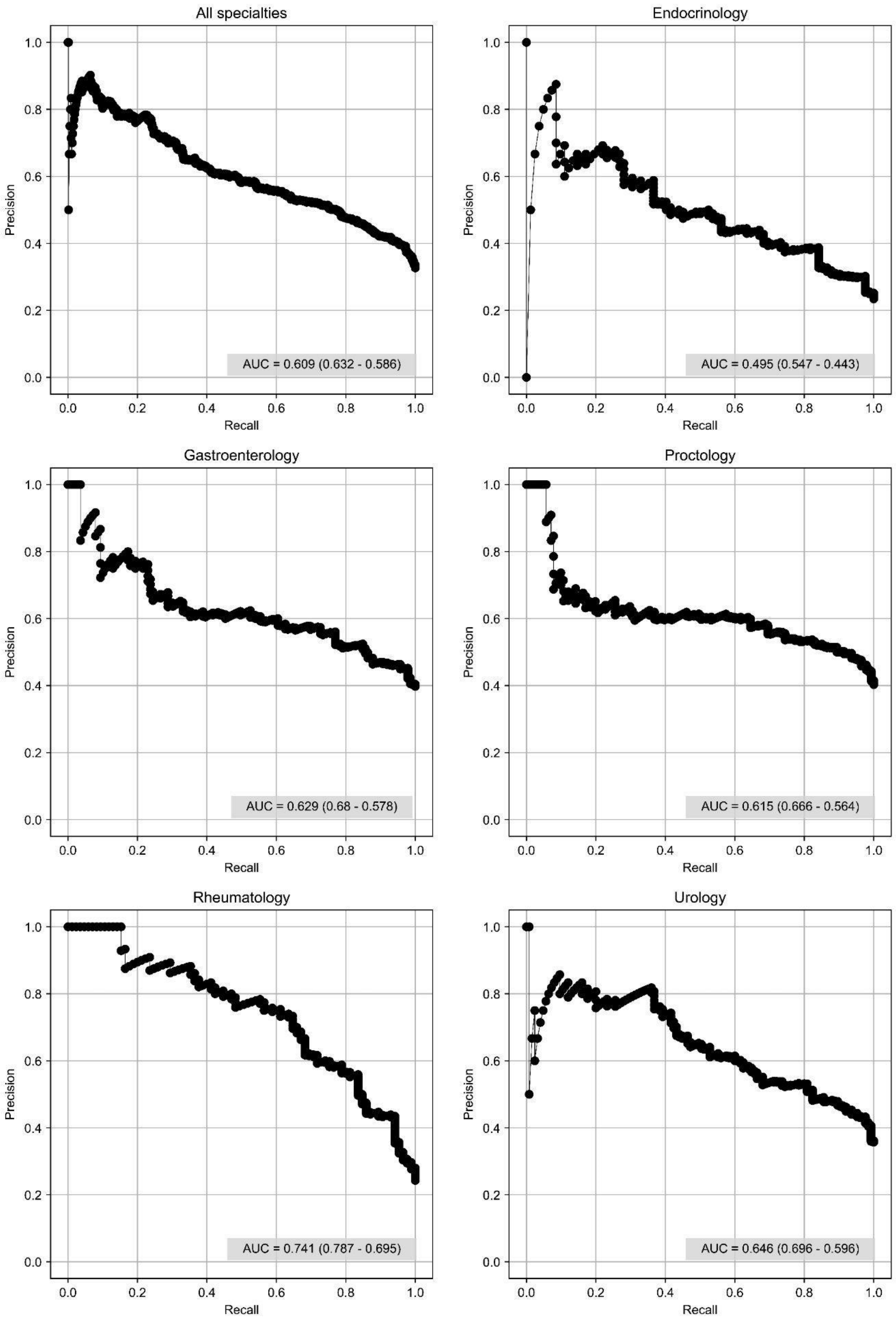

© 2025 Vergara PO et al. *JAMA Network Open*.

eFigure 3. Top 10 features (terms) with the highest predictive weight SHAP Analysis for the final AI model, by specialty. Terms translated from Portuguese.

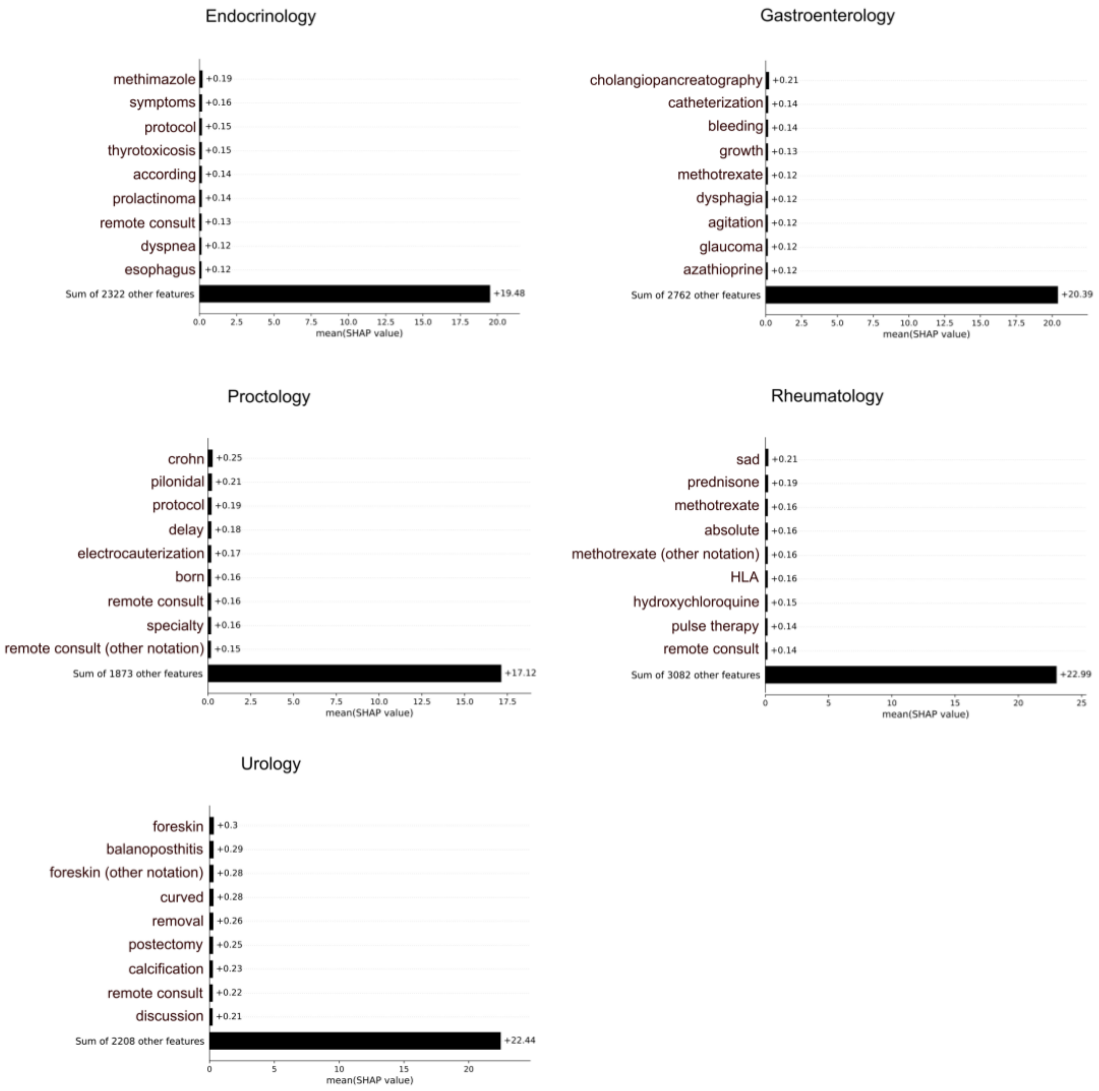

© 2025 Vergara PO et al. *JAMA Network Open*.

eFigure 4. Examples of Referrals with the Highest (A) and Lowest (B) Probability of Approval in the Dataset. Terms translated from Portuguese.

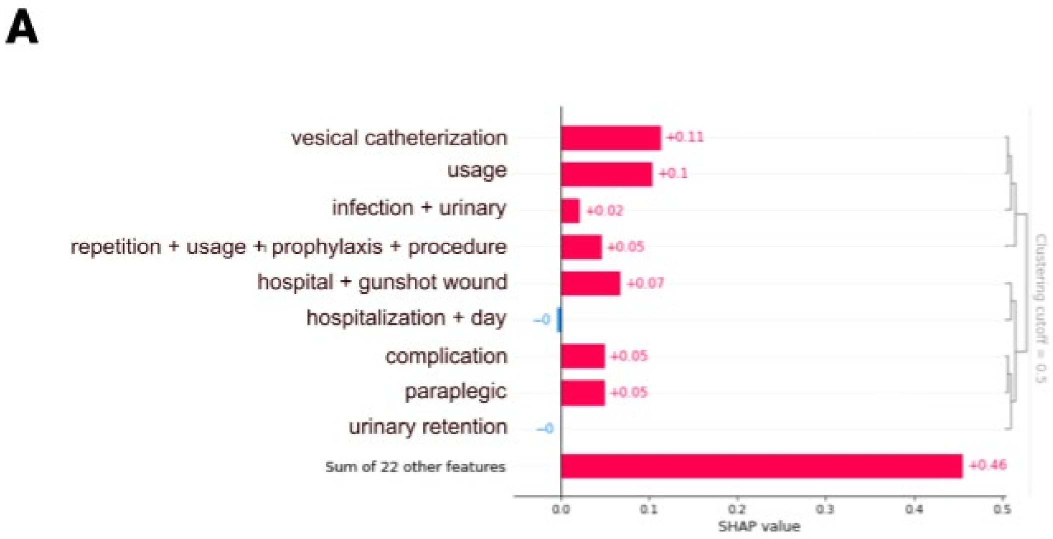

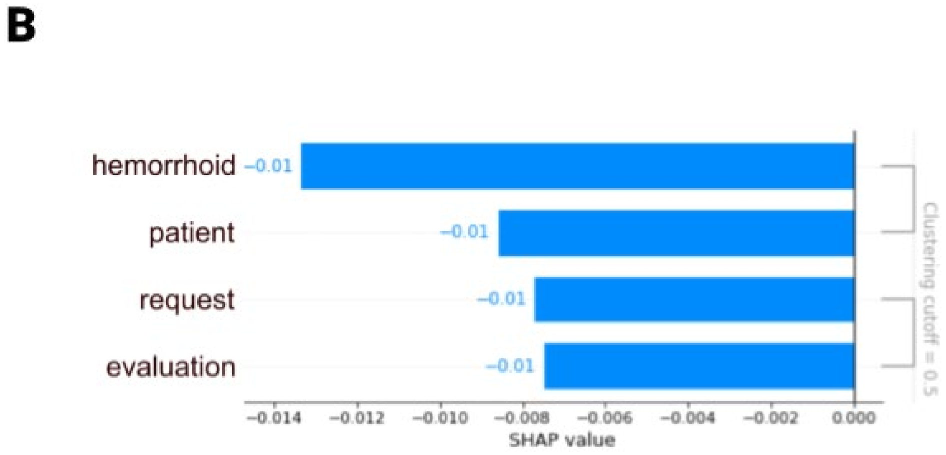

© 2025 Vergara PO et al. *JAMA Network Open*.

eTable 1. Area under the curve for the artificial intelligence model in general and according to specialties

Specialties	Mean (95% CI)
**All specialties**	0.765 (0.742 - 0.788)
**Endocrinology**	0.765 (0.708 - 0.822)
**Gastroenterology**	0.726 (0.673 - 0.779)
**Proctology**	0.726 (0.674 - 0.778)
**Rheumatology**	0.876 (0.833 - 0.919)
**Urology**	0.774 (0.724 - 0.823)

AI model: Artificial Intelligence model.

© 2025 Vergara PO et al. *JAMA Network Open*.

eTable 2. Net reclassification index (NRI) between AI model and current regulation methods.

Specialties	Net reclassification index (95% CI)	p value
**All specialties**	19.4 (16.2 - 22.5)	<0.001
**Endocrinology**	21.5 (14.4 - 28.3)	<0.001
**Gastroenterology**	-0.9 (-7.5 - 6)	0.80
**Proctology**	15.5 (8.2 - 22.6)	<0.001
**Rheumatology**	46.0 (39.2 - 52.1)	<0.001
**Urology**	15.1 (8 - 22)	<0.001

© 2025 Vergara PO et al. *JAMA Network Open*.

eTable 3. Positive and negative predictive values of AI model and current regulation methods.

	AI model	Current	Difference	p-value
**Positive predictive value**	**Mean (95% CI)**	**Mean (95% CI)**	**Mean (95% CI)**	
**All specialties**	0.568 (0.526 - 0.609)	0.397 (0.370 - 0.424)	0.145 (0.021 - 0.263)	0.02
**Endocrinology**	0.438 (0.346 - 0.533)	0.311 (0.255 - 0.373)	0.127 (-0.020 - 0.267)	0.09
**Gastroenterology**	0.613 (0.523 - 0.696)	0.574 (0.505 - 0.640)	0.039 (-0.075 - 0.152)	0.51
**Proctology**	0.604 (0.499 - 0.701)	0.437 (0.381 - 0.49.5)	0.167 (0.054 - 0.277)	0.01
**Rheumatology**	0.592 (0.495 - 0.682)	0.264 (0.218 - 0.317)	0.328 (0.180 - 0.456)	<0.001
**Urology**	0.590 (0.505 - 0.671)	0.445 (0.386 - 0.505)	0.145 (0.021 - 0.262)	0.02
**Negative predictive value**				
**All specialties**	0.782 (0.758 - 0.805)	0.871 (0.837 - 0.899)	-0.089 (-0.119 - -0.058)	<0.001
**Endocrinology**	0.853 (0.803 - 0.892)	0.922 (0.860 - 0.959)	-0.069 (-0.123 - -0.015)	0.01
**Gastroenterology**	0.714 (0.653 - 0.768)	0.844 (0.778 - 0.894)	-0.130 (-0.207 - -0.051)	<0.001
**Proctology**	0.662 (0.604 - 0.717)	0.758 (0.639 - 0.848)	-0.096 (-0.181 - -0.009)	0.03
**Rheumatology**	0.902 (0.859 - 0.934)	0.895 (0.780 - 0.956)	-0.007 (-0.059 - 0.045)	0.79
**Urology**	0.784 (0.725 - 0.834)	0.917 (0.840 - 0.960)	-0.133 (-0.198 - -0.067)	<0.001

© 2025 Vergara PO et al. *JAMA Network Open*.

eTable 4. Absolute results of the artificial intelligence model and current regulation methods.

	Current				AI model			
**Specialties**	**True positive**	**False negative**	**False positive**	**True negative**	**True positive**	**False negative**	**False positive**	**True negative**
**All specialties**	513	59	778	400	310	262	235	943
**Endocrinology**	73	9	161	107	46	36	59	209
**Gastroenterology**	116	23	86	125	73	66	46	165
**Proctology**	126	15	162	47	52	89	34	175
**Rheumatology**	80	5	222	43	61	24	42	223
**Urology**	118	7	147	78	78	47	54	171

© 2025 Vergara PO et al. *JAMA Network Open*.
