# Supplementary material for: Accuracy of Artificial Intelligence for Gatekeeping in Referrals to Specialized Care
Source: JAMA Netw Open. 2025 Jun 3;8(6):e2513285. doi: 10.1001/jamanetworkopen.2025.13285 (PMC12134955; doi:10.1001/jamanetworkopen.2025.13285)
Supplement: Supplement 2. — Data Sharing Statement [file jamanetwopen-e2513285-s002.pdf]

Data Sharing Statement

Vergara. Accuracy of Artificial Intelligence for Gatekeeping in Referrals to Specialized Care. *JAMA Netw Open*. Published June 02, 2025. doi:10.1001/jamanetworkopen.2025.13285

Data
Data available: No

Additional Information
Explanation for why data not available: Sharing Statement: Data will not be publicly shared, but the authors may be contacted with inquiries that will be discussed with the Rio Grande do Sul and Porto Alegre public health administrations.
